# Bacteriocins Targeting Gram-Negative Phytopathogenic Bacteria: Plantibiotics of the Future

**DOI:** 10.3389/fmicb.2020.575981

**Published:** 2020-09-18

**Authors:** William M. Rooney, Ray Chai, Joel J. Milner, Daniel Walker

**Affiliations:** ^1^Plant Science Group, School of Life Sciences, Institute of Molecular, Cell and Systems Biology, University of Glasgow, Glasgow, United Kingdom; ^2^College of Medical, Veterinary and Life Sciences, Institute of Infection, Immunity and Inflammation, University of Glasgow, Glasgow, United Kingdom

**Keywords:** bacteriocins, Gram-negative bacteria, phytopathogenic bacteria, plant disease, plant disease management, food security, crops

## Abstract

Gram-negative phytopathogenic bacteria are a significant threat to food crops. These microbial invaders are responsible for a plethora of plant diseases and can be responsible for devastating losses in crops such as tomatoes, peppers, potatoes, olives, and rice. Current disease management strategies to mitigate yield losses involve the application of chemicals which are often harmful to both human health and the environment. Bacteriocins are small proteinaceous antibiotics produced by bacteria to kill closely related bacteria and thereby establish dominance within a niche. They potentially represent a safer alternative to chemicals when used in the field. Bacteriocins typically show a high degree of selectivity toward their targets with no off-target effects. This review outlines the current state of research on bacteriocins active against Gram-negative phytopathogenic bacteria. Furthermore, we will examine the feasibility of weaponizing bacteriocins for use as a treatment for bacterial plant diseases.

## Introduction

By 2050 the global population is predicted to surpass 9 billion requiring food production to increase by 70%, equivalent to 127 × 10^15^ kcal (Cole et al., 2018). Major food crops suffer from a lack of genetic diversity allowing pathogens and pests to rapidly spread throughout fields and devastate crops, causing yield losses of up to 32% (Oerke and Dehne, 2004).

Gram-negative bacterial phytopathogens are an important contributor to crop losses due to plant disease (Mansfield et al., 2012). For example, *Pseudomonas syringae* pv. *actinidiae*, the causal agent of the kiwifruit canker pandemic, triggered enormous damage to the New Zealand economy (Vanneste et al., 2013) depreciating the land value of orchards growing the popular kiwifruit variety Hort16A from 300,000 to 46,000 USD per hectare (Vanneste, 2017). Enterobacterial soft rot phytopathogens such as *Pectobacterium* and *Dickeya* spp. are collectively responsible for diseases in potato like black leg and tuber soft rot pre- and post-harvest (Pérombelon, 2002; Toth et al., 2011). These diseases are responsible for losses of €30 m per annum in the Netherlands alone (Pérombelon, 2002; Toth et al., 2011).

Bacteriocins are proteinaceous antibiotics that are produced by both Gram-positive and Gram-negative bacteria (Cascales et al., 2007; Chavan and Riley, 2007). They target and kill related bacterial strains allowing producing strains to establish dominance within a niche (Chavan and Riley, 2007). Unlike conventional small molecule antibiotics, bacteriocins exhibit a narrow killing spectrum and cause minimal disruption to the commensal bacterial community (Chavan and Riley, 2007). A number of classification systems have been proposed to encompass the diversity of bacteriocins (Heng and Tagg, 2006; Chavan and Riley, 2007; Cotter et al., 2013). The classification of Chavan and Riley (2007) is based on size, splitting the bacteriocins into three groups; small peptide bacteriocins of <10 kDa, colicin-like bacteriocins (CLBs) which are multidomain proteins of 25–80 kDa and tailocins, which are large phage-like multimeric protein assemblies. This review focuses on the latter two of these groups as there is a dearth of information on small peptide bacteriocins active against phytopathogenic bacteria. In addition, in this review we cover an additional group, the lectin-like bacteriocins (LLBs), which although they fall within the size range of CBLs, are mechanistically distinct. We also provide examples of some orphan bacteriocins.

Bacteriocins have been identified in a number of important plant pathogenic bacterial genera including *Xanthomonas*, *Pseudomonas*, *Pectobacterium*, and *Agrobacterium* (Holtsmark et al., 2008; Grinter et al., 2012a). These include many important pathogens of crops such as rice, banana, potato, olives, peppers and tomatoes (Mansfield et al., 2012). In this review, we aim to outline the present landscape of research into bacteriocin plantibiotics (biological agents which selectively kill plant pathogenic bacteria) and discuss the practicalities of exploiting them to remedy plant disease.

## Colicin-Like Bacteriocins

CLBs are multi-domain proteins that possess a modular domain structure usually consisting of translocation, receptor binding and cytotoxic domains. The translocation domain typically incorporates, or consists of an intrinsically disordered region (IDR) at the extreme N-terminus of the protein, which is first to cross the outer membrane during uptake (Behrens et al., 2020). To target a specific bacterial species, CLBs often parasitize existing nutrient uptake pathways involving TonB dependent transporters (TBDTs). These TBDTs are frequently involved with the uptake of iron siderophores and other metal chelate complexes, such as vitamin B_12_, from the environment (Michel-Briand and Baysse, 2002; Cascales et al., 2007). For most CLBs the IDR and translocation domains facilitate the import of bacteriocins across the outer membrane into the periplasmic space. Briefly, this is achieved by the IDR threading through the pore of an outer membrane transporter and interacting with components of the proton-motive force (PMF) responsive Ton or Tol complexes in the periplasm. Subsequently, the bacteriocin is actively pulled through the transporter in a PMF-dependent manner to cross the outer membrane (White et al., 2017). Methods of killing mediated by CLB cytotoxic domains include endonuclease activity (DNase, tRNase, and rRNase), depolarization of the inner-membrane, and inhibition of peptidoglycan synthesis (Michel-Briand and Baysse, 2002; Cascales et al., 2007).

**FIGURE 1:**
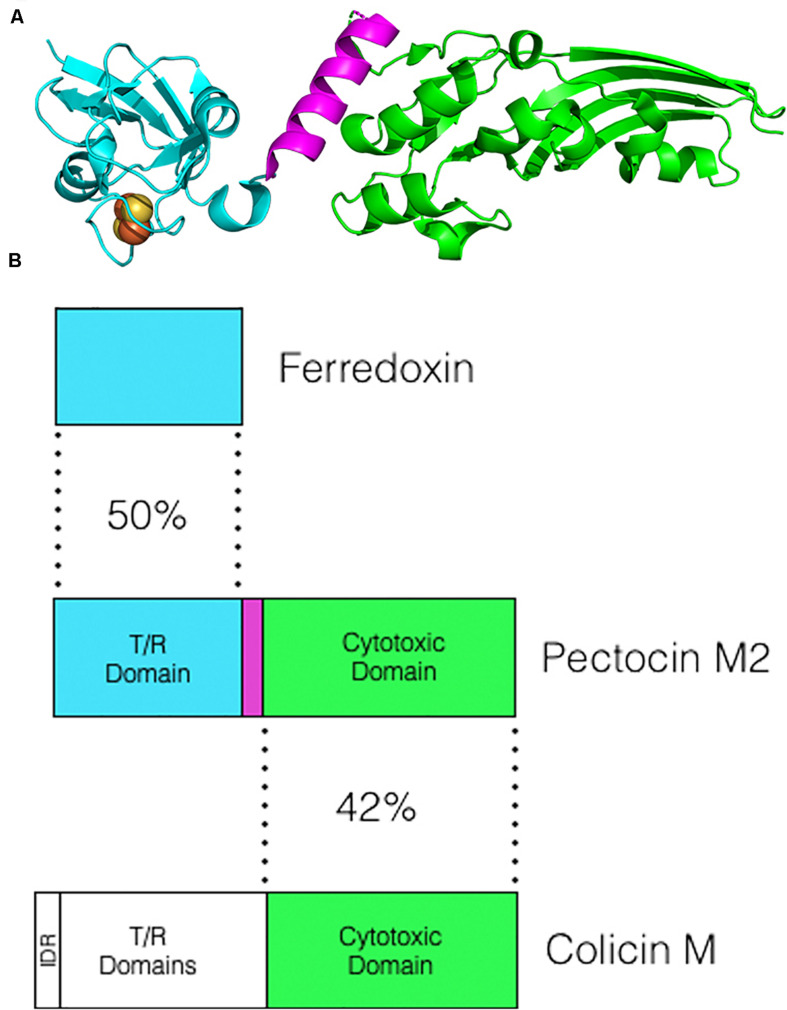
Structure and homology of the ferredoxin containing pectocin M2. **(A)** The crystal structure of pectocin M2 (PDB:4N58). The ferredoxin domain is in cyan, the linker region in purple and the colicin M-like cytotoxic domain in green. The iron sulfur cluster located in the ferredoxin domain is represented as yellow and orange spheres. **(B)** The N-terminal domain of pectocin M2 (ZP_03825528) shares homology with spinach ferredoxin (1704156A). The C-terminal domain which is separated from the ferredoxin domain by a short helical linker shares homology with the lipid II-cleaving cytotoxic domain of colicin M (WP_000449474). Numbers shown are percentage identities calculated using the Needleman-Wunsch algorithm (Needle program) from EBI.

In *Pectobacterium carotovorum*, three CLB nucleases termed carocins have been characterized. Two of the carocins, S1K (40 kDa) and carocin D (91 kDa) are DNases while the third, S2 (85 kDa) is a tRNAse (Chuang et al., 2007; Chan et al., 2009, 2011; Roh et al., 2010; Atanaskovic et al., 2020). In addition, two pectobacterial CLBs, pectocins M1 and M2 (both 29 kDa) have been characterized that possess cytotoxic domains homologous to that of colicin M and have been shown to similarly target lipid II (Grinter et al., 2012b). Cleavage of lipid II by colicin M-like bacteriocins results in inhibition of peptidoglycan biosynthesis and cell death (Harkness and Ölschläger, 1991). Interestingly, pectocin M1 and M2 lack an IDR at their N-termini and instead contain a single globular domain N-terminal to the cytotoxic domain that is homologous in both sequence and structure to plant ferredoxin (Figure 1; Grinter et al., 2012b, 2014). Like plant ferredoxin, these CLBs also contain a 2Fe-2S iron sulfur cluster and as subsequent research has shown, have evolved to parasitize an existing ferredoxin uptake system utilized by *Pectobacterium* spp. to acquire iron from its plant hosts. Uptake of ferredoxin is mediated by the TBDT FusA and the TonB-like protein FusB which work in concert to translocate ferredoxin into the periplasm (Grinter et al., 2016; Wojnowska and Walker, 2020). FusB acts both in removal of the plug from the lumen of FusA and directly binding to ferredoxin mediating its active translocation across the outer membrane via the lumen of FusA (Wojnowska and Walker, 2020). Within the periplasm, the processing protease FusC cleaves ferredoxin in two specific locations releasing its iron into the periplasm (Mosbahi et al., 2018). Competition assays with spinach ferredoxin and killing assays under iron limiting conditions show that ferredoxin-containing bacteriocins are translocated using the same pathway (Grinter et al., 2012b). Bioinformatic analysis has revealed another putative pectobacterial bacteriocin, pectocin P (35 kDa), that also contains a ferredoxin domain (Grinter et al., 2012b). However, the cytotoxic domain of pectocin P shares structural homology to lysozyme implying that uptake using the ferredoxin domain can be utilized as a general translocation pathway to deliver cytotoxic proteins into the periplasm. Lastly, two CLBs from *P. syringae* have been reported, syringacin M (30 kDa), which shares homology with colicin M, and a nuclease bacteriocin, S_*E9a*_ (64 kDa) related to pyocin S2 (Grinter et al., 2012c; Hockett et al., 2017). Unlike the colicin M-like pectocins M1 and M2, syringacin M does possess an N-terminal IDR and so likely has an uptake mechanism that is similar to the well-characterized colicins from *E. coli* (Grinter et al., 2012c).

Analysis of mutations in bacteria grown in the presence of bacteriocins suggest that resistance usually results from changes in the bacteriocin receptor (Cascales et al., 2007; Inglis et al., 2016). However, the development of resistance in the wild is still poorly understood and it may also depend on additional factors involving the receptor. For example, in iron limiting conditions, resistance to pyocin S2 is subject to negative selection as its receptor is required for the uptake of iron (Inglis et al., 2016). As these receptors are typically involved in processes that are important for competition and cell survival, resistant strains tend to be less fit and show reduced pathogenicity in some environments.

## Lectin-Like Bacteriocins

LLBs are a distinct family of protein antibiotics found in *Pseudomonas*, *Burkholderia*, and *Xanthomonas* species (Ghequire et al., 2012a, 2013a). The hallmark of LLBs is the presence of monocot mannose-binding lectin (MMBLs) domains. MMBLs are expanded in both plants and animals and play a primitive defensive role against pests and pathogens (Ghequire et al., 2012b). LLBs possess at least 1 MMBL domain containing conserved QxDxNxVxYx sequences that constitute a carbohydrate-binding pocket. These MMBLs are instrumental in defining the selectivity of LLBs by enabling the docking onto D-rhamnose-containing lipopolysaccharide (LPS) on the cell surface (Ghequire et al., 2013b; McCaughey et al., 2014).

**FIGURE 2:**
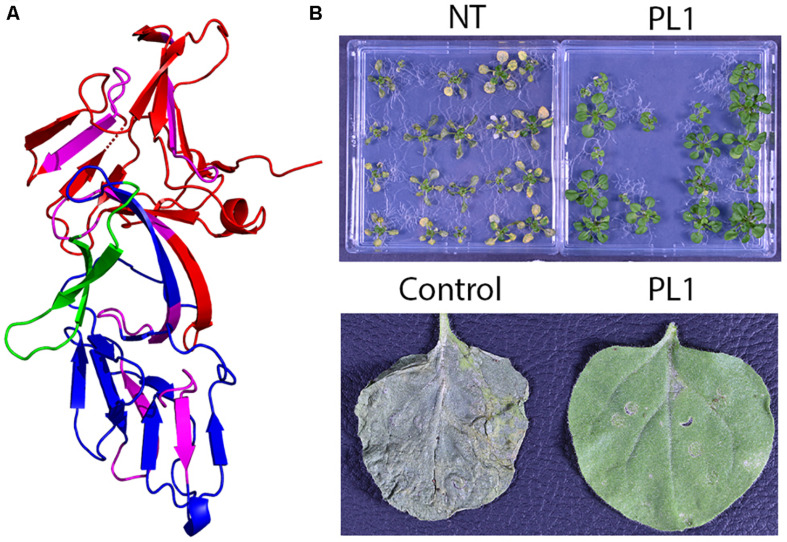
LLBs can be provide robust disease resistance against *P. syringae*. **(A)** Structure of the LLB PL1 (PDB:4GC2). The N- and C-terminal MMBL domains are shown in red and blue, respectively, and C-terminal extension is shown in green. Within these domains the QxDxNxVxYx sugar binding motifs are shown in purple. The C-terminal extension is predicted to play a role in the cytotoxic action of PL1 by disrupting the function of BamA. **(B)** Non-transgenic (NT) vs. transgenic expression of PL1 in both Arabidopsis seedlings (upper panel) and *Nicotiniana benthamiana* leaves (lower panel) provides robust resistance against strains of *P. syringae* that are susceptible to PL1. These images were adapted from Rooney et al. (2019).

Our current understanding of LLBs arises predominantly from the study of pyocin L1 and putidacin L1 (PL1) isolated from *P. aeruginosa* and *P. putida*, respectively (Parret et al., 2003; McCaughey et al., 2014). PL1 (30 kDa) harbors 2 MMBL domains (Figure 2A) and phylogenetic analyses of the N- and C-terminal MMBL domains suggest distinct functions in LPS docking (Ghequire et al., 2013b). The N-terminal MMBL domains diverge substantially implying their importance in selectivity, whereas the C-terminal MMBL domains tightly cluster suggesting that their primary function are to bind to carbohydrates with high affinity (Ghequire et al., 2018b). Intriguingly, it was recently reported that LLBs containing 1 MMBL exhibit anti-microbial activity against Pseudomonads (Ghequire and De Mot, 2019). Although little is known about these bacteriocins isolated from soil- and plant-associated bacteria, their MMBLs share homology with the N-terminal MMBL domains of putidacin L1-type LLBs (Ghequire and De Mot, 2019).

Resistance to LLBs can arise from changes to LPS structure by susceptible bacteria (Ghequire et al., 2013b; McCaughey et al., 2014). However, LPS binding does not fully explain the selective nature of LLBs (Ghequire et al., 2013b). An exhaustive genetic study of resistant bacterial isolates identified novel changes in a surface-exposed extracellular loop of the outer membrane protein BamA (Ghequire et al., 2018a). BamA is a critical component of the β-barrel assembly machinery responsible for the chaperoning and insertion of β-barrel proteins into the outer membrane (Noinaj et al., 2017). Sequence alignments comparing PL1 sensitive and resistant strains identified the amino acid sequences of loop 6 of BamA as a genetic determinant of PL1 susceptibility. This was elegantly demonstrated when a “resistant” allele of BamA successfully rescued a PL1-sensitive strain from PL1-mediated killing *in vitro* (Ghequire et al., 2018a).

## Tailocins

Tailocins are headless phage tail-like bacteriocins consisting of 8–14 individual components, including a sheath, core and baseplate (Ghequire et al., 2015b; Scholl, 2017). A producing cell releases 100s of particles and sometimes one particle is sufficient to eliminate a target cell (Scholl, 2017). Although tailocins from phytopathogenic bacteria share a high degree of similarity with contractile tail phages derived from *Myoviridae*, they have evolved independently, and represent an expansive group of protein complexes playing critical ecological roles like biofilm formation (Ghequire et al., 2015b; Turnbull et al., 2016). Tailocin-mediated killing occurs in two steps. Firstly, the tail fibers selectively bind to LPS of a target cell (Michel-Briand and Baysse, 2002). In turn, the sheath contracts and punctures the cell envelope, depolarizing the cell membrane and resulting in cell death (Scholl, 2017).

Tailocins are produced by a range of bacteria including *Pseudomonads* and *Pectobacterium* spp. (Nguyen et al., 2001; Hockett et al., 2015). Remarkably, tailocins from *P. syringae* have evolved independently of those of *P. aeruginosa* and do not share the same evolutionary ancestor (Ghequire and De Mot, 2015a; Hockett et al., 2015). This likely reflects the different environmental niches that *P. aeruginosa* and *P. syringae* occupy. Intriguingly, diversification and expansion of the tailocin family in *P. syringae* is driven by localized recombination of tailocin genes like those encoding the tail fibers (Baltrus et al., 2019).

Identification and genetic dissection of two distinct tailocins from *P. chlororaphis* has unmasked the robust competitive advantage tailocins provide in heterogeneous biofilms and the rhizosphere (Dorosky et al., 2017, 2018). Notably, *P. fluorescens* SF4c harbors a tailocin targeting *X. vesicatoria*, the causal agent of bacterial spot disease in tomatoes (Príncipe et al., 2018). In the case of *Pectobacterium* there are two highly similar tailocins, carotovoricin (Ctv)Er and CGE originating from *P. carotovorum IAM 1068* and *P. carotovorum* CGE234-M403, respectively (Yamada et al., 2006). Indeed, both carotovoricins are identical apart from two 26 bp inverted repeats within and downstream of the tail fiber gene, which differentiates their killing spectrum (Nguyen et al., 2001). Notably, strains of *Pectobacterium* harboring CtvCGE are sold in Japan, under the name “Biokeeper” to manage bacterial soft rot infections in potatoes (Chuang et al., 1999).

Classically, tailocin tolerance arises from alterations in the LPS, enabling the targets to evade tailocin recognition (Scholl, 2017; Kandel et al., 2020). A recent study postulates that bacteria can persist in environments containing sub-lethal concentrations of tailocins (Kandel et al., 2020). Persistence is not a heritable genetic trait, and likely reflects heterogeneity of gene expression within a clonal population influenced by factors such as starvation and metabolic activity (Kandel et al., 2020). Persistence allows bacteria to bypass mutations which incur fitness costs providing the selective pressure is transient.

## Orphan Bacteriocins and Plantibiotics

Several bacteriocins from phytopathogens do not display homology with other well-characterized classes. For example, *X. campestris* pv. *glycines* 8ra produces a bacteriocin called glycinecin A (55 kDa), which is unusually encoded by two genes (Heu et al., 2001). Interestingly, although glycinecin A can be produced recombinantly in *E. coli*, active bacteriocin is only obtained when *glyA* and *glyB* are co-expressed in the same cell; active bacteriocin cannot be reconstituted by combining the two separately expressed polypeptides *in vitro* (Heu et al., 2001). There are two bacteriocins identified from *X. campestris* and *X. perforans*, glycinecin R, and BCN-A, containing Rhs repeats (pfam05593) which share homology with the toxin complex of the insect pathogen *Photorhabdus* (Roh et al., 2008; Marutani-Hert et al., 2020). The mode of action for these *Xanthomonas* bacteriocins are unknown.

The production of a very narrow spectrum bacteriocin-like substance agrocin 84 (1.4 kDa) by some non-pathogenic strains of *Agrobacterium tumefaciens* has been characterized and exploited to control crown gall diseases caused by pathogenic strains of *A. tumefaciens* (Kerr and Htay, 1974; Ellis et al., 1979). This small nucleotide antibiotic represses leucyl-tRNA synthetase activity (Tate et al., 1979; Kim et al., 2006). Remarkably, *A. tumefaciens* strains that successfully evolve resistance against agrocin 84 become non-pathogenic (Kerr, 1980). The success of agrocin 84 as a strategy to control crown gall disease resulted in the development of an agrocin 84-producing *A. tumefaciens* strain which was successfully trademarked and sold under the name Nogall by Bio-care Technology (Jones et al., 1988).

## Applications of Bacteriocins and Future Perspectives

The control of bacterial phytopathogens in agriculture often relies on the application of chemicals containing copper or antibiotics, notably streptomycin. These often have detrimental impacts on human health and the environment and their long term success as a control measure can be limited by the development of resistance (Sundin and Bender, 1993; Damalas and Eleftherohorinos, 2011). For example, streptomycin treatment has been used extensively in orchards to mitigate diseases like fire blight and citrus greening. However, widespread applications of antibiotics in a field context has the potential to create reservoirs of resistance that can potentially transfer from plant pathogenic bacteria into bacterial pathogens of clinical importance (Norelli et al., 2003; McKenna, 2019).

A major driver of the success of the insecticidal protein *Bacillus thuringiensis* (Bt) toxin has been its high degree of target selectivity and its ease of expression *in planta*. Bacteriocins share similar characteristics. In both clinical and agricultural contexts this is highly advantageous as their use would be expected to cause minimal disruption to the microbiome. Like Bt toxins, bacteriocins can be expressed in plants or directly applied to crops. Nomad Biosciences have neatly illustrated the feasibility of expressing bacteriocins (LLBs and CLBs) in several plant species (Schulz et al., 2015; Paškevičius et al., 2017; Schneider et al., 2018). Furthermore, there is little evidence of bacteriocin toxicity in various animal models (Behrens et al., 2017). Bacteriocins are naturally produced by environmental bacteria, it is thought that they have limited toxicity toward humans, animals or benign environmental bacterial species and some bacteriocins are already classified as generally regarded as safe for use in food preservation (Schulz et al., 2015). As we have recently shown, bacteriocins can be expressed transgenically *in planta* to provide resistance against *P. syringae*. Expression of PL1 in two model plant species provided a strong resistance phenotype in plants challenged with several unrelated PL1-sensitive *P. syringae* field isolates (Figure 2B; Rooney et al., 2019) with bacterial titres in PL1 transgenic lines 1.5 log-units lower than in non-transgenic controls (Rooney et al., 2019).

Non-GM-based protocols for bacteriocin-based control measures include examples where non-pathogenic but bacteriocin producing strains of bacteria have been directly applied to crops, for example, Nogall and Biokeeper (Jones et al., 1988; Chuang et al., 1999). Alternatively, treatments using bacteriocins as a direct application to crops have shown promise in laboratory conditions against olive knot disease and bacterial spot disease of tomato (Lavermicocca et al., 2002; Príncipe et al., 2018). One potential issue in utilizing bacteriocins as a direct treatment is the requirement for large scale bacteriocin production. This maybe technically difficult for multi-component bacteriocins (e.g., tailocins) but should not be a problem for LLBs and CLBs where successful production *in planta* has already been demonstrated (Schulz et al., 2015; Paškevičius et al., 2017; Rooney et al., 2019).

The organization of bacteriocins into functional domains enables them to be readily engineered, providing a potential route for producing further variants by domain swapping to create new chimeric bacteriocins with altered target activities and modes of killing (Lukacik et al., 2012). Similarly, for CLBs appropriate domain swapping could yield chimeric bacteriocins for which there is no immunity protein-based resistance in the targeted bacterial species (Akutsu et al., 1989). For tailocins, the exchange of tail fibers has already been shown to produce novel chimeras (Baltrus et al., 2019).

Despite the discovery and characterization of bacteriocins from phytopathogens, there is limited proof of a competitive advantage for the producing strain *in vivo*. Evidence suggests that soluble bacteriocins like CLBs function in the apoplastic space (endophytic fitness) whereas tailocins function in rhizosphere communities (epiphytic fitness) (Dorosky et al., 2018; Li et al., 2020). However, *in vitro* data suggests bacteriocins could work in concert in a conditionally redundant manner (Hockett et al., 2017).

Overall, bacteriocins represent an under-utilized resource of disease control. In the age of metagenomics, this can be easily remedied by the swift identification and characterization of new bacteriocins. This would allow bacteriocins to be rapidly deployed against current and emerging threats to important food crops.

## Author Contributions

WR, RC, JM, and DW contributed to the original manuscript and the editorial changes. All authors contributed to the article and approved the submitted version.

## Conflict of Interest

The University of Glasgow has filed a patent on the transgenic expression of PL1 in plants with WR, JM, and DW listed as inventors (PCT/EP2018/057826). The remaining author declares that the research was conducted in the absence of any commercial or financial relationships that could be construed as a potential conflict of interest.

## Abbreviations

CLB: Colicin-like bacteriocin
GM: genetically modified
IDR: intrinsically disordered region
LLB: lectin-like bacteriocin
LPS: lipopolysaccharide
MMBL: monocot-mannose binding lectin
PL1: putidacin L1
TBDT: TonB-dependent transporter.

## References

[B1] Akutsu A. Masaki H. Ohta T. (1989). Molecular structure and immunity specificity of colicin E6, an evolutionary intermediate between E-group colicins and cloacin DF13. *J. Bacteriol.* 171 6430–6436. 10.1128/jb.171.12.6430-6436.1989 2687234 PMC210531

[B2] Atanaskovic I. Mosbahi K. Sharp C. Housden N. G. Kaminska R. Walker D. (2020). Targeted killing of *Pseudomonas aeruginosa* by Pyocin G occurs via the hemin transporter Hur. *J. Mol. Biol.* 432 3869–3880. 10.1016/j.jmb.2020.04.020 32339530 PMC7322526

[B3] Baltrus D. A. Clark M. Smith C. Hockett K. L. (2019). Localized recombination drives diversification of killing spectra for phage-derived syringacins. *ISME J.* 13 237–249. 10.1038/s41396-018-0261-3 30171255 PMC6331570

[B4] Behrens H. M. Lowe E. D. Gault J. Housden N. G. Kaminska R. Weber T. M. (2020). Pyocin S5 import into *Pseudomonas aeruginosa* reveals a generic mode of bacteriocin transport. *mBio* 11:e3230-19. 10.1128/mBio.03230-19 PMC7064778 32156826

[B5] Behrens H. M. Six A. Walker D. Kleanthous C. (2017). The therapeutic potential of bacteriocins as protein antibiotics. *Emerg. Top. Life Sci.* 1 65–74. 10.1042/etls20160016 PMC7243282 33525816

[B6] Cascales E. Buchanan S. K. Duche D. Kleanthous C. Lloubes R. Postle K. (2007). Colicin biology. *Microbiol. Mol. Biol. Rev.* 71 158–229. 17347522 10.1128/MMBR.00036-06 PMC1847374

[B7] Chan Y. C. Wu H. P. Chuang D. Y. (2009). Extracellular secretion of Carocin S1 in *Pectobacterium carotovorum* subsp. *carotovorum* occurs via the type III secretion system integral to the bacterial flagellum. *BMC Microbiol.* 9:181. 10.1186/1471-2180-9-181 19712460 PMC2744703

[B8] Chan Y. C. Wu J. L. Wu H. P. Tzeng K. C. Chuang D. Y. (2011). Cloning, purification, and functional characterization of Carocin S2, a ribonuclease bacteriocin produced by *Pectobacterium carotovorum*. *BMC Microbiol.* 11:99. 10.1186/1471-2180-11-99 21569432 PMC3120645

[B9] Chavan M. A. Riley M. A. (2007). *Molecular Evolution of Bacteriocins in Gram-Negative Bacteria. In*: *Bacteriocins Ecology and Evolution.* Berlin: Springer, 19–43.

[B10] Chuang D. Y. Chien Y. C. Wu H. P. (2007). Cloning and expression of the *Erwinia carotovora* subsp. *carotovora* gene encoding the low-molecular-weight bacteriocin carocin *S*1. *J. Bacteriol.* 189 620–626. 10.1128/jb.01090-06 17071754 PMC1797388

[B11] Chuang D. Y. Kyeremeh A. G. Gunji Y. Takahara Y. Ehara Y. Kikumoto T. (1999). Identification and cloning of an *Erwinia carotovora* subsp. *carotovora* bacteriocin regulator gene by insertional mutagenesis. *J. Bacteriol.* 181 1953–1957. 10.1128/jb.181.6.1953-1957.1999 10074096 PMC93602

[B12] Cole M. B. Augustin M. A. Robertson M. J. Manners J. M. (2018). The science of food security. *NPJ Sci. Food* 2:14. 10.1038/s41538-018-0021-9 PMC6550266 31304264

[B13] Cotter P. D. Ross R. P. Hill C. (2013). Bacteriocins-a viable alternative to antibiotics? *Nat. Rev. Microbiol.* 11 95–105. 10.1038/nrmicro2937 23268227

[B14] Damalas C. A. Eleftherohorinos I. G. (2011). Pesticide exposure, safety issues, and risk assessment indicators. *Int. J. Environ. Res. Public Health* 8 1402–1419. 10.3390/ijerph8051402 21655127 PMC3108117

[B15] Dorosky R. J. Pierson L. S. Pierson E. A. (2018). *Pseudomonas chlororaphis* produces multiple R-tailocin particles that broaden the killing spectrum and contribute to persistence in rhizosphere communities. *Appl. Environ. Microbiol.* 84:e01230-18. 10.1128/AEM.01230-18 30030224 PMC6121977

[B16] Dorosky R. J. Yu J. M. Pierson L. S. Pierson E. A. (2017). *Pseudomonas chlororaphis* produces two distinct R-tailocins that contribute to bacterial competition in biofilms and on roots. *Appl. Environ. Microbiol.* 83:e00706-17. 10.1128/AEM.00706-17 28526791 PMC5514687

[B17] Ellis J. G. Kerr A. Van Montagu M. Schell J. (1979). Agrobacterium: genetic studies on agrocin 84 production and the biological control of crown gall. *Physiol. Plant Pathol.* 15 311–316. 10.1016/0048-4059(79)90082-1

[B18] Ghequire M. G. K. De Canck E. Wattiau P. Van Winge I. Loris R. Coenye T. (2013a). Antibacterial activity of a lectin-like *Burkholderia cenocepacia* protein. *MicrobiologyOpen* 2 566–575. 10.1002/mbo3.95 23737242 PMC3831624

[B19] Ghequire M. G. K. De Mot R. (2015a). The tailocin tale: peeling off phage tails. *Trends Microbiol.* 23 587–590. 10.1016/j.tim.2015.07.011 26433692

[B20] Ghequire M. G. K. De Mot R. (2019). LlpB represents a second subclass of lectin-like bacteriocins. *Microbial Biotechnol.* 12 567–573. 10.1111/1751-7915.13373 30702207 PMC6465234

[B21] Ghequire M. G. K. Dillen Y. Lambrichts I. Proost P. Wattiez R. De Mot R. (2015b). Different ancestries of R tailocins in rhizospheric *Pseudomonas* isolates. *Genome Biol. Evol.* 7 2810–2828. 10.1093/gbe/evv184 26412856 PMC4684702

[B22] Ghequire M. G. K. Garcia-Pino A. Lebbe E. K. M. Spaepen S. Loris R. de Mot R. (2013b). Structural determinants for activity and specificity of the bacterial toxin LlpA. *PLoS Pathog.* 9:e1003199. 10.1371/journal.ppat.1003199 23468636 PMC3585409

[B23] Ghequire M. G. K. Li W. Proost P. Loris R. De Mot R. (2012b). Plant lectin-like antibacterial proteins from phytopathogens *Pseudomonas syringae* and *Xanthomonas citri*. *Environ. Microbiol. Rep.* 4 373–380. 10.1111/j.1758-2229.2012.00331.x 23760822

[B24] Ghequire M. G. K. Loris R. De Mot R. (2012a). MMBL proteins: from lectin to bacteriocin. *Biochem. Soc. Trans.* 40 1553–1559. 10.1042/bst20120170 23176516

[B25] Ghequire M. G. K. Öztürk B. De Mot R. (2018a). Lectin-like bacteriocins. *Front. Microbiol.* 9:2706. 10.3389/fmicb.2018.02706 30483232 PMC6240691

[B26] Ghequire M. G. K. Swings T. Michiels J. Buchanan S. K. De Mot R. (2018b). Hitting with a BAM: selective killing by lectin-like bacteriocins. *mBio* 9:e02138-17.) 10.1128/mBio.02138-17 PMC5874912 29559575

[B27] Grinter R. Josts I. Mosbahi K. Roszak A. W. Cogdell R. J. Bonvin A. M. J. J. (2016). Structure of the bacterial plant-ferredoxin receptor FusA. *Nat. Commun.* 7:133308. 10.1038/ncomms13308 27796364 PMC5095587

[B28] Grinter R. Josts I. Zeth K. Roszak A. W. Mccaughey L. C. Cogdell R. J. (2014). Structure of the atypical bacteriocin pectocin M2 implies a novel mechanism of protein uptake. *Mol. Microbiol.* 93 234–246. 10.1111/mmi.12655 24865810 PMC4671253

[B29] Grinter R. Milner J. Walker D. (2012a). Bacteriocins active against plant pathogenic bacteria. *Biochem. Soc. Trans.* 40 1498–1502. 10.1042/bst20120206 23176505

[B30] Grinter R. Milner J. Walker D. (2012b). Ferredoxin containing bacteriocins suggest a novel mechanism of iron uptake in *Pectobacterium* spp. *PLoS One* 7:e33033. 10.1371/journal.pone.0033033 22427936 PMC3302902

[B31] Grinter R. Roszak A. W. Cogdell R. J. Milner J. J. Walker D. (2012c). The crystal structure of the lipid II-degrading bacteriocin syringacin M suggests unexpected evolutionary relationships between colicin M-like bacteriocins. *J. Biol. Chem.* 287 38876–38888. 10.1074/jbc.m112.400150 22995910 PMC3493929

[B32] Harkness R. E. Ölschläger T. (1991). The biology of colicin M. *FEMS Microbiol. Lett.* 8 27–41. 10.1111/j.1574-6968.1991.tb04955.x 1931137

[B33] Heng N. C. K. Tagg J. R. (2006). What’s in a name? Class distinction for bacteriocins. *Nat. Rev. Microbiol.* 4:160 10.1038/nrmicro1273-c2

[B34] Heu S. Oh J. Kang Y. Ryu S. Cho S. K. Cho Y. (2001). gly Gene cloning and expression and purification of glycinecin A, a bacteriocin produced by *Xanthomonas campestris* pv. *glycines* 8ra. *Appl. Environ. Microbiol.* 67 4105–4110. 10.1128/aem.67.9.4105-4110.2001 11526012 PMC93136

[B35] Hockett K. L. Clark M. Scott S. Baltrus D. A. (2017). Conditionally redundant bacteriocin targeting by *Pseudomonas* syringae. *bioRxiv [Preprint].* 10.1101/167593

[B36] Hockett K. L. Renner T. Baltrus D. A. (2015). Independent co-option of a tailed bacteriophage into a killing complex in *Pseudomonas*. *mBio* 6:e00452-15. 10.1128/mBio.00452-15 26265717 PMC4542187

[B37] Holtsmark I. Eijsink V. G. H. Brurberg M. B. (2008). Bacteriocins from plant pathogenic bacteria. *FEMS Microbiol. Lett.* 280 1–7. 10.1111/j.1574-6968.2007.01010.x 18070073

[B38] Inglis R. F. Scanlan P. Buckling A. (2016). Iron availability shapes the evolution of bacteriocin resistance in *Pseudomonas aeruginosa*. *ISME J.* 10 2060–2066. 10.1038/ismej.2016.15 26905630 PMC4883653

[B39] Jones D. A. Ryder M. H. Clare B. G. Farrand S. K. Kerr A. (1988). Construction of a Tra - deletion mutant of pAgK84 to safeguard the biological control of crown gall. *MGG Mol. Gen. Genet.* 212 207–214. 10.1007/bf00334686

[B40] Kandel P. P. Baltrus D. A. Hockett K. L. (2020). *Pseudomonas* can survive tailocin killing via persistence-like and heterogenous resistance mechanisms. *J. Bacteriol.* 202:e00142-20. 10.1128/JB.00142-20 32312747 PMC7283598

[B41] Kerr A. (1980). Biological control of crown gall through production of Agrocin 84. *Plant Dis.* 64 25–30.

[B42] Kerr A. Htay K. (1974). Biological control of crown gall through bacteriocin production. *Physiol. Plant Pathol.* 4 37–40. 10.1016/0048-4059(74)90042-3

[B43] Kim J. G. Byoung K. P. Sung-Uk K. Doil C. Baek H. N. Jae S. M. (2006). Bases of biocontrol: sequence predicts synthesis and mode of action of agrocin 84, the Trojan Horse antibiotic that controls crown gall. *Proc. Natl. Acad. Sci.* 103 8846–8851. 10.1073/pnas.0602965103 16731618 PMC1482666

[B44] Lavermicocca P. Lisa Lonigro S. Valerio F. Evidente A. Visconti A. (2002). Reduction of olive knot disease by a bacteriocin from *Pseudomonas syringae* pv. *ciccaronei*. *Appl. Environ. Microbiol.* 68 1403–1407. 10.1128/aem.68.3.1403-1407.2002 11872493 PMC123734

[B45] Li J. Z. Zhou L. Y. Peng Y. L. Fan J. (2020). *Pseudomonas* bacteriocin syringacin M released upon desiccation suppresses the growth of sensitive bacteria in plant necrotic lesions. *Microbial Biotechnol.* 13 134–147. 10.1111/1751-7915.13367 30672132 PMC6922522

[B46] Lukacik P. Barnard T. J. Keller P. W. Chaturvedi K. S. Seddiki N. Fairman J. W. (2012). Structural engineering of a phage lysin that targets Gram-negative pathogens. *Proc. Natl. Acad. Sci. U.S.A.* 109 9857–9862. 10.1073/pnas.1203472109 22679291 PMC3382549

[B47] Mansfield J. Genin S. Magori S. Citovsky V. Sriariyanum M. Ronald P. (2012). Top 10 plant pathogenic bacteria in molecular plant pathology. *Mol. Plant Pathol.* 13 614–629. 10.1111/j.1364-3703.2012.00804.x 22672649 PMC6638704

[B48] Marutani-Hert M. Hert A. P. Tudor-Nelson S. M. Preston J. F. Minsavage G. V. Stall R. E. (2020). Characterization of three novel genetic loci encoding bacteriocins associated with *Xanthomonas perforans*. *PLoS One* 15:e0233301. 10.1371/journal.pone.0233301 32469926 PMC7259588

[B49] McCaughey L. C. Grinter R. Josts I. Roszak A. W. Waløen K. I. Cogdell R. J. (2014). Lectin-like bacteriocins from *Pseudomonas* spp. Utilise D-Rhamnose Containing Lipopolysaccharide as a Cellular Receptor. *PLoS Pathog.* 10:e1003898. 10.1371/journal.ppat.1003898 24516380 PMC3916391

[B50] McKenna M. (2019). Antibiotics set to flood Florida’s troubled orange orchards. *Nature* 567 302–303. 10.1038/d41586-019-00878-874 30890811

[B51] Michel-Briand Y. Baysse C. (2002). The pyocins of *Pseudomonas aeruginosa*. *Biochimie* 26 329–336. 10.1016/s0300-9084(02)01422-0 12423794

[B52] Mosbahi K. Wojnowska M. Albalat A. Walker D. (2018). Bacterial iron acquisition mediated by outer membrane translocation and cleavage of a host protein. *Proc. Natl. Acad. Sci. U.S.A.* 115 6840–6845. 10.1073/pnas.1800672115 29891657 PMC6042079

[B53] Nguyen H. A. Tomita T. Hirota M. Kaneko J. Hayashi T. Kamio Y. (2001). DNA inversion in the tail fiber gene alters the host range specificity of carotovoricin Er, a phage-tail-like bacteriocin of phytopathogenic *Erwinia carotovora* subsp. *carotovora Er*. *J. Bacteriol.* 183 6274–6281. 10.1128/jb.183.21.6274-6281.2001 11591670 PMC100113

[B54] Noinaj N. Gumbart J. C. Buchanan S. K. (2017). The β-barrel assembly machinery in motion. *Nat. Rev. Microbiol*. 15 197–204. 10.1038/nrmicro.2016.191 28216659 PMC5455337

[B55] Norelli J. L. Jones A. L. Aldwinckle H. S. (2003). Fire blight management in the twenty-first century: using new technologies that enhance host resistance in apple. *Plant Dis.* 87 756–765. 10.1094/pdis.2003.87.7.756 30812883

[B56] Oerke E. C. Dehne H. W. (2004). Safeguarding production—losses in major crops and the role of crop protection. *Crop Protect.* 23 275–285. 10.1016/j.cropro.2003.10.001

[B57] Parret A. H. A. Schoofs G. Proost P. De Mot R. (2003). Plant lectin-like bacteriocin from a rhizosphere-colonizing *Pseudomonas* isolate. *J. Bacteriol.* 185 897–908. 10.1128/jb.185.3.897-908.2003 12533465 PMC142807

[B58] Paškevičius Š Starkevič U. Misiūnas A. Vitkauskienë A. Gleba Y. Ražanskienė A. (2017). Plant-expressed pyocins for control of *Pseudomonas aeruginosa*. *PLoS One* 12:e0185782. 10.1371/journal.pone.0185782 28973027 PMC5626474

[B59] Pérombelon M. C. M. (2002). Potato diseases caused by soft rot erwinias: an overview of pathogenesis. *Plant Pathol.* 51 1–12. 10.1046/j.0032-0862.2001.short

[B60] Príncipe A. Fernandez M. Torasso M. Godino A. Fischer S. (2018). Effectiveness of tailocins produced by prin in controlling the bacterial-spot disease in tomatoes caused by *Xanthomonas vesicatoria*. *Microbiol. Res.* 213 94–102. 10.1016/j.micres.2018.05.010 29853172

[B61] Roh E. Heu S. Moon E. (2008). Genus-specific distribution and pathovar-specific variation of the glycinecin R gene homologs in Xanthomonas genomes. *J. Microbiol.* 46 681–686. 10.1007/s12275-008-0209-9 19107397

[B62] Roh E. Park T. H. Lee S. Ryu S. Oh C. S. Rhee S. (2010). Characterization of a new bacteriocin, carocin D, from *Pectobacterium carotovorum* subsp. *carotovorum Pcc*21. *Appl. Environ. Microbiol.* 76 7541–7549. 10.1128/aem.03103-09 20870796 PMC2976183

[B63] Rooney W. M. Grinter R. W. Correia A. Parkhill J. Walker D. C. Milner J. J. (2019). Engineering bacteriocin-mediated resistance against the plant pathogen *Pseudomonas syringae*. *Plant Biotechnol. J.* 18 1296–1306. 10.1111/pbi.13294 31705720 PMC7152609

[B64] Schneider T. Hahn-Löbmann S. Stephan A. Schulz S. Giritch A. Naumann M. (2018). Plant-made *Salmonella* bacteriocins salmocins for control of *Salmonella* pathovars. *Sci. Rep.* 8:4078. 10.1038/s41598-018-22465-9 29511259 PMC5840360

[B65] Scholl D. (2017). Phage tail–like bacteriocins. *Ann. Rev. Virol.* 4 453–467. 10.1146/annurev-virology-101416-041632 28961412

[B66] Schulz S. Stephan A. Hahn S. Bortesi L. Jarczowski F. Bettmann U. (2015). Broad and efficient control of major foodborne pathogenic strains of *Escherichia coli* by mixtures of plant-produced colicins. *Proc. Natl. Acad. Sci. U.S.A.* 112 E5454–E5460. 26351689 10.1073/pnas.1513311112 PMC4603501

[B67] Sundin G. W. Bender C. L. (1993). Ecological and genetic analysis of copper and streptomycin resistance in *Pseudomonas syringae* pv. *syringae*. *Appl. Environ. Microbiol.* 59 1018–1024. 10.1128/aem.59.4.1018-1024.1993 8476279 PMC202231

[B68] Tate M. E. Murphy P. J. Roberts W. P. Kerr A. (1979). Adenine N6-substituent of agrocin 84 determines its bacteriocin-like specificity [21]. *Nature* 280 697–699. 10.1038/280697a0 471050

[B69] Toth I. K. van der Wolf J. M. Saddler G. Lojkowska E. Hélias V. Pirhonen M. (2011). Dickeya species: an emerging problem for potato production in Europe. *Plant Pathol.* 60 385–399. 10.1111/j.1365-3059.2011.02427.x

[B70] Turnbull L. Toyofuku M. Hynen A. L. Kurosawa M. Pessi G. Petty N. K. (2016). Explosive cell lysis as a mechanism for the biogenesis of bacterial membrane vesicles and biofilms. *Nat. Commun.* 7:11220. 10.1038/ncomms11220 27075392 PMC4834629

[B71] Vanneste J. L. (2017). The scientific, economic, and social impacts of the New Zealand outbreak of bacterial canker of Kiwifruit (*Pseudomonas syringae* pv. *actinidiae)*. *Ann. Rev. Phytopathol.* 55 377–399. 10.1146/annurev-phyto-080516-035530 28613977

[B72] Vanneste J. L. Yu J. Cornish D. A. Tanner D. J. Windner R. Chapman J. R. (2013). Identification, virulence, and distribution of two biovars of *Pseudomonas syringae* pv. *actinidiae in New Zealand*. *Plant Dis.* 97 708–719. 10.1094/pdis-07-12-0700-re 30722585

[B73] White P. Joshi A. Rassam P. Housden N. G. Kaminska R. Goult J. D. (2017). Exploitation of an iron transporter for bacterial protein antibiotic import. *Proc. Natl. Acad. Sci. U.S.A.* 114 12051–12056. 10.1073/pnas.1713741114 29078392 PMC5692591

[B74] Wojnowska M. Walker D. (2020). FusB energises import across the outer membrane through direct interaction with its ferredoxin substrate. *bioRxiv[Preprint].* 10.1101/749960 PMC7593965 33109756

[B75] Yamada K. Hirota M. Niimi Y. Nguyen H. A. Takahara Y. Kamio Y. (2006). Nucleotide sequences and organization of the genes for carotovoricin (Ctv) from *Erwinia carotovora* indicate that Ctv evolved from the same ancestor as *Salmonella* typhi prophage. *Biosci. Biotechnol. Biochem.* 70 2236–2247. 10.1271/bbb.60177 16960352

